# The Molecular Interplay between Human Coronaviruses and Autophagy

**DOI:** 10.3390/cells10082022

**Published:** 2021-08-07

**Authors:** Ankit Shroff, Taras Y. Nazarko

**Affiliations:** Department of Biology, Georgia State University, Atlanta, GA 30303, USA; ashroff@gsu.edu

**Keywords:** autophagy, autophagosome, lysosome, autolysosome, coronavirus, SARS-CoV, SARS-CoV-2, MERS-CoV, HCoV-NL63, HCoV-OC43

## Abstract

Coronavirus disease 2019 (COVID-19), caused by a new severe acute respiratory syndrome coronavirus 2 (SARS-CoV-2), has instantaneously emerged as a worldwide pandemic. However, humans encountered other coronaviruses in the past, and they caused a broad range of symptoms, from mild to life-threatening, depending on the virus and immunocompetence of the host. Most human coronaviruses interact with the proteins and/or double-membrane vesicles of autophagy, the membrane trafficking pathway that degrades and recycles the intracellular protein aggregates, organelles, and pathogens, including viruses. However, coronaviruses often neutralize and hijack this pathway to complete their life cycle. In this review, we discuss the interactions of human coronaviruses and autophagy, including recent data from SARS-CoV-2-related studies. Some of these interactions (for example, viral block of the autophagosome–lysosome fusion), while being conserved across multiple coronaviruses, are accomplished via different molecular mechanisms. Therefore, it is important to understand the molecular interplay between human coronaviruses and autophagy for developing efficient therapies against coronaviral diseases.

## 1. Introduction

Autophagy is a cytoplasmic membrane trafficking process, which was initially discovered as a recycling pathway for bulk cytosol and, later, for specific intracellular components [1]. It is essential for cells in periods of starvation and other stresses for degradation of misfolded and aggregated proteins [2], as well as damaged and surplus organelles [3]. Apart from degradation of the cell’s own constituents, autophagy is also involved in host response to microbial infections [4]. In a nutshell, macroautophagy, the most studied type of autophagy discussed in this review, constitutes a formation of the double-membrane vesicle, the autophagosome. The autophagosome is decorated with microtubule-associated protein 1 light chain 3 alpha or beta (MAP1LC3A/B, hereafter LC3) on both inner and outer membranes. LC3 binds various receptor proteins, such as sequestosome 1 (SQSTM1; also known as p62), that bridge the select cytoplasmic components destined for degradation with the growing autophagic membrane. The completed autophagosomes carry the enclosed substrates for degradation in the lytic compartment, the lysosome. The formation of the autophagosome starts from the nucleation that involves beclin 1 (BECN1). This is followed by the action of many autophagy-related (ATG) proteins, such as ATG5, that elongate the cup-shaped autophagic membrane until it is sealed into the completed double-membrane autophagosome [5]. Autophagosome formation is often followed by a fusion of the autophagosome with the endosome, creating an intermediate compartment, the amphisome. Then, both autophagosome and amphisome can fuse with the lysosome, a process that also depends on BECN1 [6]. After this terminal fusion, the inner membrane of the autophagosome, together with the sequestered material inside, is degraded by the lysosomal hydrolases. The vesicle formed by the fusion of the autophagosome/amphisome and lysosome is called the autolysosome. The macromolecules, organelles, and pathogens are decomposed in the autolysosomes to building blocks that are exported back to the cytosol for reuse, while components of the pathogens are also presented to T cells as antigens to activate the adaptive immune responses.

Coronaviruses are positive strand RNA viruses that can utilize the host cellular machinery for their replication and cause human diseases. The recent coronavirus disease 2019 (COVID-19) has developed into the pandemic and affected a significant number of people worldwide [7]. The causative agent has been named SARS-CoV-2, as it bears significant similarity to the coronavirus that causes severe acute respiratory syndrome (SARS), SARS-CoV [8]. The severity of COVID-19 in some patients also resembles Middle East respiratory syndrome (MERS) caused by the related coronavirus, MERS-CoV [9]. These large viruses, enveloped by a lipid bilayer, enter host cells via binding to the cell surface receptors [10]. This binding is followed by the endocytosis of viral particles that might proceed via multiple routes [11]. Then, the particle membrane fuses with the endosomal membrane and nucleocapsid enters the cytoplasm of the host cell with subsequent uncoating of the viral genome. Next, the genomic RNA of coronaviruses uses cellular translation machinery to synthesize viral replicase, the RNA-dependent RNA polymerase, and subgenomic RNAs use the same to synthesize structural and accessory proteins. The replication proteins form a complex, which is localized inside the double-membrane vesicles. Since these vesicles resemble autophagosomes, many studies investigated the relationship between the coronaviral particles or their individual proteins and autophagosomes [12]. Below, we review known interactions of human coronaviruses with autophagy, a fascinating pathway that recycles intracellular material via the double-membrane vesicular intermediates.

## 2. Interactions between Human Coronaviruses and Autophagy

**Figure 1:**
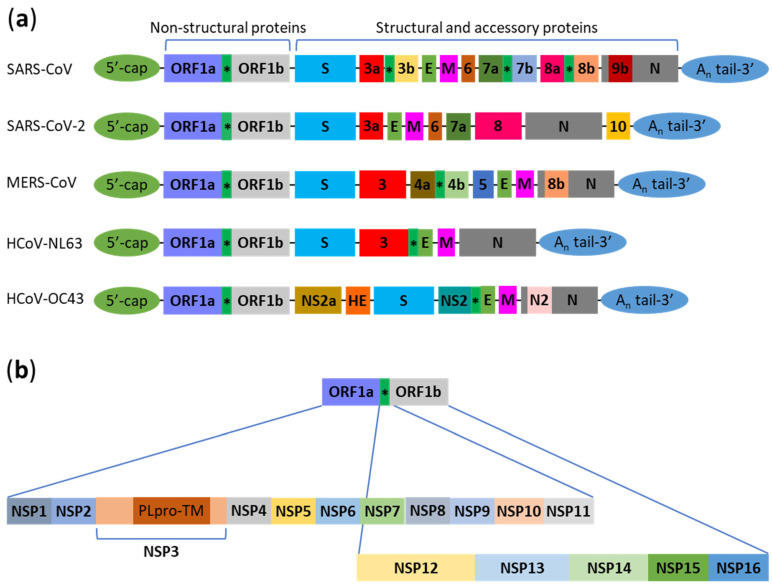
Genome organization of human coronaviruses that were used in autophagy studies. (**a**) Genomes of SARS-CoV, SARS-CoV-2, MERS-CoV, HCoV-NL63, and HCoV-OC43 (not to scale) [12,17]; (**b**) non-structural proteins (NSPs) of human coronaviruses produced by cleavage of polyproteins that are encoded by ORF1a and ORF1b [18,19]. ORF and NSP symbols are in bold. * Frameshift.

Human coronaviruses (HCoV) belong to the genera *Alphacoronavirus* and *Betacoronavirus*. The genus *Alphacoronavirus* includes HCoV-229E and HCoV-NL63. The genus *Betacoronavirus* contains human coronaviruses in three lineages: A, B, and C. The lineage A includes HCoV-OC43 and HCoV-HKU1, lineage B contains SARS-CoV and SARS-CoV-2, while lineage C includes MERS-CoV [12]. HCoV-229E, HCoV-NL63, HCoV-OC43, and HCoV-HKU1 are known to cause the common cold in most people. However, their infection could result in a life-threatening pneumonia or bronchiolitis in immunocompromised individuals, infants, and elderly people [13]. On the other hand, SARS-CoV, SARS-CoV-2, and MERS-CoV infection causes flu-like symptoms and pneumonia in most individuals. In severe cases, their infection results in fatal respiratory failure and acute respiratory distress syndrome [14,15]. The interactions with autophagy have been studied for five human coronaviruses to date: SARS-CoV, SARS-CoV-2, MERS-CoV, HCoV-NL63, and HCoV-OC43 (Figure 1a). Since LC3 is an established marker of autophagosomes [16], many studies tested the levels and localization of this protein. The increased levels of the autophagosome-bound LC3 type II (LC3-II), which is a lipidated form of the cytosolic LC3-I protein, and the increased number of LC3-positive puncta suggest the accumulation of autophagosomes, which can be either due to autophagy induction or a downstream block of the autophagic flux (e.g., block of the autophagosome–lysosome fusion that was reported in several studies, described below).

### 2.1. SARS-CoV

**Figure 2:**
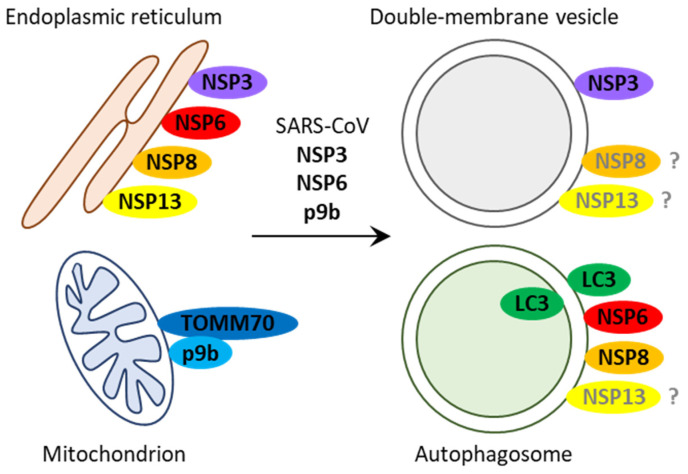
Interactions between SARS-CoV and autophagy. SARS-CoV, its endoplasmic reticulum-associated non-structural proteins, NSP3 and NSP6, and mitochondria-localized accessory protein, p9b, induce accumulation of double-membrane vesicles marked by NSP3 and autophagosomes tagged with LC3, NSP6, and NSP8. Protein symbols are in bold.

The first severe HCoV studied with respect to its interaction with autophagy was SARS-CoV. Various studies analyzed the effect of individual proteins of SARS-CoV on the host cellular machinery to understand the mechanisms involved in this host–pathogen interaction. The ORF1a of SARS-CoV encodes a polyprotein with several non-structural proteins (NSPs) (Figure 1b). The NSP3 contains the papain-like protease (PLpro) and transmembrane (TM) domains [18,20]. The transfection of the PLpro-TM coding sequence in human embryonic kidney (HEK293T) cells increases the levels of LC3-II [19]. Similar results were obtained with NSP6 that localizes to the endoplasmic reticulum (ER), induces formation of LC3-positive autophagosomes, and partially co-localizes with them in Chinese hamster ovary (CHO) cells [21]. Interestingly, the autophagosomes generated by NSP6 in African green monkey kidney epithelial (Vero) cells are significantly smaller (≤0.5 µm) than autophagosomes induced by starvation [22]. Another study demonstrated that NSP8 co-localizes pairwise with NSP2, NSP3, and LC3 in Vero E6 cells infected with SARS-CoV [23]. In a different study involving the infection of Vero E6 cells with SARS-CoV, the double-membrane vesicles (0.2–0.3 µm in size) were associated with the ER and mitochondria [24]. Immunostaining for SARS-CoV proteins on ultrathin sections of cells showed that NSP3, NSP8, and NSP13 localize to these double-membrane structures and the ER. However, the authors did not observe any co-localization between NSP3 and LC3 or the late endosomal/lysosomal marker, lysosomal-associated membrane protein 1 (LAMP1) [24]. Collectively, these findings suggest that SARS-CoV and some of its ER-localized non-structural proteins (NSP3 and NSP6) can induce the accumulation of double-membrane vesicles, including autophagosomes (Figure 2). However, while some viral proteins (NSP6 and NSP8) mark autophagosomes, NSP3 does not, leaving a possibility that the SARS-CoV replication complex might be associated with more than one type of double-membrane vesicle.

Further studies with SARS-CoV implied its potential interaction with mitophagy, the selective autophagy of mitochondria. The SARS-CoV 9b protein localizes to mitochondria and induces an increased LC3-II/LC3-I ratio in adenocarcinomic human alveolar basal epithelial (A549) cells. As expected, this increase is ATG5 dependent (ATG5 is known to be involved in LC3-I lipidation and autophagosome formation). It also correlates with the increased number of LC3-positive autophagosomes and their association with mitochondria [25]. Relatedly, Pichlmair and colleagues showed that both the SARS-CoV and SARS-CoV-2 9b proteins interact with the mitophagy regulator, translocase of outer mitochondrial membrane 70 (TOMM70), and cause mitochondrial dysfunction in A549 cells [26]. Therefore, in addition to non-selective autophagy, SARS coronaviruses might also affect mitophagy.

In another study, Gilch and colleagues showed that a proteasome inhibitor, MG-132, strongly reduces SARS-CoV titers in Vero E6 cells due to inhibition of the cysteine protease, m-calpain [27]. In mouse embryonic fibroblasts (MEFs) expressing the human receptor of SARS-CoV, angiotensin I-converting enzyme 2 (hACE2), and infected with SARS-CoV, MG-132 treatment results in a complete absence of viral titers. At the same time, the knockout (KO) of the *Atg5* gene in hACE2-expressing MEFs does not affect viral titers, suggesting that the SARS-CoV replication in these cells is independent of Atg5-mediated autophagy. However, treatment of the SARS-CoV-infected *Atg5* KO MEFs expressing hACE2 with MG-132 causes a less pronounced decrease in viral titers. Therefore, the effect of MG-132 partially depends on Atg5-mediated autophagy [27]. Together, these findings demonstrate that some accessory proteins of SARS-CoV, such as mitochondrial p9b, are also able to induce autophagosome accumulation (Figure 2). However, while relying on m-calpain for its replication, SARS-CoV might not require autophagosomes for this. Instead, the induction of autophagy (in addition to inhibition of m-calpain) with MG-132 [27] might help human cells to get rid of the virus.

### 2.2. SARS-CoV-2

#### 2.2.1. Early Interactions with Autophagy

The interactions of SARS-CoV-2 with autophagy might occur at different stages of the viral life cycle. While it is now well established that ACE2 is a primary receptor that mediates SARS-CoV-2 entry into host cells, studies on its co-receptors are still ongoing. Integrins are the major class of co-receptors known to assist in viral entry. Ivarsson and colleagues showed that integrin subunit beta 3 (ITGB3) contains the LC3-interacting region (LIR) that, upon phosphorylation of the upstream and/or downstream phosphosites, can bind to various Atg8 family proteins, including LC3 [28]. This in vitro work suggests a potential interaction of the SARS-CoV-2 particles with autophagic machinery as soon as they become attached to the cell surface of host cells, but further studies are required to test this hypothesis in vivo.

The viral spike (S) protein mediates the initial contact of SARS-CoV-2 particles with the ACE2 receptor. A recent study found that the GU-rich RNA region of the SARS-CoV-2 S gene induces secretion of the pro-inflammatory cytokines, interleukin 1 beta (IL1B), tumor necrosis factor (TNF), and interleukin 6 (IL6), by human macrophages [29]. The authors further observed that a combination of GU-rich RNA expression with vitamin D3 treatment leads to a modulation of autophagy. Specifically, this combination causes a significant decrease in the levels of autophagic receptor/substrate, SQSTM1, coupled with conversion of LC3-I to LC3-II, indicating induction of autophagy. To confirm the involvement of autophagy in these processes, Spector and colleagues inhibited it using siRNA against the core autophagic gene, *ATG5*. This inhibition resulted in decreased secretion of IL1B, but not TNF and IL6, indicating that autophagy is involved in the secretion of IL1B produced in response to GU-rich viral RNA [29]. Therefore, targeting autophagic machinery could be one of the ways to modulate the cytokine storm observed during SARS-CoV-2 infection.

#### 2.2.2. Protein M Promotes Mitophagy

In agreement with the previous study, SARS-CoV-2 infection did stimulate autophagy (specifically, mitophagy), as judged by an increased number of LC3-positive autophagosomes and elevated LC3-II levels, but decreased levels of the mitochondrial markers TOMM20 and translocase of inner mitochondrial membrane 23 (TIMM23), in Vero E6, human hepatocellular carcinoma (Huh-7), and colorectal adenocarcinoma (Caco-2) cells [30]. Interestingly, Jin and colleagues identified the viral membrane (M) protein behind these effects because the expression of a single M protein could recapitulate them, in addition to decreased SQSTM1 levels. Excitingly, their study provided a glimpse into the molecular mechanism of mitophagy induction by SARS-CoV-2. Via its interaction with the mitochondrial Tu translation elongation factor (TUFM), M protein co-localizes with the mitochondria. Here, it acts as a heterologous mitophagy receptor by recruiting (using its canonical LIR motif) LC3-II to mitochondria and causing their degradation. While M protein lacking LIR still localizes to mitochondria, it recruits reduced amounts of LC3-II, leading to an intermediate number of LC3-positive dots and intermediate levels of LC3-II protein in cells. Notably, both M protein expression and SARS-CoV-2 infection cause re-localization of LC3 to mitochondria, indicating that this might be a physiologically relevant mechanism. Moreover, this mechanism may have important immunological consequences as M protein (but, to a lesser extent, M without LIR) affects transcriptional activation of interferon beta 1 (IFNB1) via DExD/H-box helicase 58 (DDX58; also known as RIG-I) and mitochondrial antiviral signaling protein (MAVS) [30]. Additionally, blocking autophagy with the phosphatidylinositol 3-kinase inhibitors, 3-methyladenine and wortmannin, or specific block of phosphatidylinositol 3-kinase catalytic subunit type 3 (PIK3C3; also known as VPS34) with Vps34-IN1 inhibitor, limits SARS-CoV-2 replication in Vero E6, Huh-7, and Caco-2 cells, and human ex vivo lung tissue culture [30,31], suggesting that the M protein-induced mitophagy might play a role in viral replication.

#### 2.2.3. Protein 8 Causes Selective Autophagy of MHC-I Molecules

In addition to the potential role of autophagy/mitophagy in suppressing the type I interferon response (see above), autophagy is also responsible for the evasion of SARS-CoV-2-infected cells from cytotoxic T lymphocytes [32]. The study of Zhang and colleagues found that SARS-CoV-2 infection downregulates the surface levels of major histocompatibility complex class Ι (MHC-Ι) molecules on hACE2-expressing HEK293T cells and lung epithelial cells from hACE2 mice. The same was true during SARS-CoV-2 (but not SARS-CoV) p8 expression in HEK293T and other cells. Moreover, p8 knockdown (KD) restored MHC-I surface levels on hACE2-expressing HEK293T cells infected with SARS-CoV-2, proving this accessory protein as a main player here. Interestingly, p8 downregulates MHC-I molecules presenting viral peptides to cytotoxic T lymphocytes via autophagy. SARS-CoV-2 p8 binds MHC-I A2 (HLA-A2) and both proteins co-localize with LC3-positive autophagosomes. Moreover, p8 also binds BECN1, which might facilitate sequestration of p8-HLA-A2 complexes into autophagosomes. The abrogation of autophagosome formation via the KD/KO of autophagic genes, such as *ATG5*, *ATG7*, *BECN1*, *GABARAP*, and *RB1CC1* (also known as *FIP200*), or blocking autophagosome turnover with lysosomal inhibitors, such as bafilomycin A1, chloroquine, and E64d/pepstatin A, restores the surface levels of HLA-A2. These rescue results confirm the vital role of the autophagy–lysosomal pathway in the p8-mediated degradation of MHC-I molecules [32].

#### 2.2.4. NSP15 Inhibits Autophagosome Formation

Unexpectedly, a global transcriptomic analysis of SARS-CoV-2-infected adenocarcinomic human airway epithelial (Calu-3) and A549 cells showed that cellular transcriptome leans towards autophagy inhibition [33]. Two potential mechanisms may be responsible for this inhibition. One mechanism could involve upregulation of autophagy inhibitors, such as a member of the RAS oncogene family, RAB5A, that operates by inducing the expression of an autophagy inhibitor, mechanistic target of rapamycin kinase (MTOR). The other is via downregulation of autophagy inducers, such as high mobility group box 1 (HMGB1), sigma non-opioid intracellular receptor 1 (SIGMAR1), and sterol regulatory element-binding transcription factor 2 (SREBF2). While this in silico analysis awaits further validation in vivo, the study on the peripheral blood mononuclear cells (PBMCs) of COVID-19 patients demonstrated that expression of the autophagy-inducing gene interferon regulatory factor 8 (*IRF8*), and several autophagy-related genes, such as *ULK1*, *ATG5*, *UVRAG*, *AMBRA1*, *PIK3C3*, and *LC3*, was significantly downregulated relative to PBMCs of healthy individuals [34]. This downregulation translated into a reduced LC3-II/LC3-I ratio. Since all the patients received hydroxychloroquine, an inhibitor of autophagosome–lysosome fusion, these results are consistent with the reduced autophagosome formation in the PBMCs of COVID-19 patients. At least one viral protein, NSP15, could be responsible for this outcome as its expression in human cervical cancer (HeLa) and HEK293T cells does abolish the formation of autophagosomes [35]. The transcriptional downregulation of autophagy by SARS-CoV-2 also received some confirmation at the protein level. The levels of core autophagic proteins, ATG5, ATG12, and GABA type A receptor-associated protein (GABARAP), were reduced in SARS-CoV-2-infected Vero E6 cells [36]. In addition, proteomic analyses of human pulmonary alveolar epithelial cells (HPAEpiCs) infected with SARS-CoV-2 showed downregulation of autophagy-related proteins [37]. Among the different ways that SARS-CoV-2 proteins affect autophagic proteins, there is also a regulation of their post-translational modifications, namely ubiquitination and phosphorylation, that was studied in A549 cells [26]. A group of autophagy-related proteins, such as MAP1LC3A, GABARAP, VPS33A, and VAMP8, is increasingly ubiquitinated upon SARS-CoV-2 infection, while a group of autophagy regulatory proteins, such as DEPTOR, RICTOR, OPTN, SQSTM1, and LAMTOR1, is highly regulated by phosphorylation in response to SARS-CoV-2 [26].

#### 2.2.5. Proteins 3a, 7a, and E Block Autophagosome Turnover

**Figure 3:**
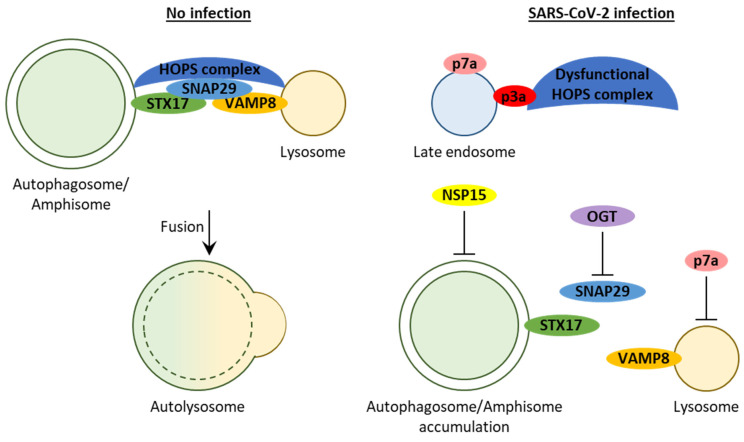
Interactions between SARS-CoV-2 and autophagy. SARS-CoV-2 inhibits autophagosome formation by NSP15 and blocks autophagosome turnover by p3a, p7a, and E protein (not shown). The p3a localizes to the late endosomes and assembles a dysfunctional HOPS complex there. This depletes HOPS complex at the autophagosomes/amphisomes and affects formation of the specific *trans*-SNARE complex between the autophagosomal SNARE, STX17, lysosomal SNARE, VAMP8, and their bridging protein, SNAP29, leading to reduced autophagosome/amphisome–lysosome fusion and accumulation of autophagosomes/amphisomes. SNAP29 is also inhibited by the host OGT enzyme. Therefore, OGT depletion can bypass the negative effect of p3a on autophagosome/amphisome–lysosome fusion. The p7a protein also localizes to late endosomes and affects the acidification of lysosomes. Protein symbols are in bold and in ovals.

Remarkably, some studies also reported elevated levels of LC3-II [36] and MAP1LC3B2 [37], and accumulation of double-membrane vesicles and fragmented mitochondria in the cytoplasm of SARS-CoV-2-infected cells [37], suggesting a block in autophagosome turnover. This block is consistent with SARS-CoV-2 infection in Calu-3 and Caco-2 cells that caused increased levels of both LC3-II and SQSTM1 [35]. Three viral proteins might be responsible for such a block: p3a, p7a, and envelope (E) protein (with p3a being the most prominent), because they were able to cause decreased autophagosome turnover (increased levels of LC3-II and SQSTM1) during ectopic expression in HEK293T, HeLa, and A549 cells [35,38,39]. Interestingly, a couple of SARS-CoV-2 proteins (NSP6 and p3a) can interact with and/or control the levels of select autophagic proteins [26]. For example, p3a increases the abundance of autophagic receptors, such as SQSTM1, NBR1, CALCOCO2 (also called NDP52), and TAX1BP1, as well as Atg8 family proteins, such as MAP1LC3A, MAP1LC3B (including LC3-II), GABARAP, and GABARAPL2. The co-accumulation of autophagic receptors and Atg8 family proteins (especially LC3-II) suggests inhibition of autophagic flux after the autophagosome formation step. Indeed, the levels of the established autophagic substrate, apolipoprotein B (APOB), were also increased [26] further supporting the potential role of p3a in blocking autophagosome turnover. In summary, SARS-CoV-2 infection might inhibit autophagy at different stages in different cells via diverse regulatory mechanisms (Figure 3).

#### 2.2.6. Protein 3a Affects Autophagosome/Amphisome–Lysosome Fusion

Several recent studies uncovered the detailed mechanism of how SARS-CoV-2 p3a blocks autophagy [26,35,38,39]. Interestingly, autophagy induction by starvation, INK128, or rapamycin cannot rescue the autophagy block in the p3a-expressing HeLa, A549, or HEK293T cells [35,38,39]. These results were consistent with the dramatic accumulation of puncta of several autophagosomal markers, such as LC3, ATG14, WIPI2, and STX17, in cells expressing p3a under both normal and starvation conditions [35,38,39]. Moreover, the LC3 puncta in the p3a-expressing cells co-localized with RAB7A-positive late endosomes and partially co-localized with LAMP1-positive late endosomes/lysosomes, but did not co-localize with LAMP2A-positive lysosomes, supporting normal amphisome but not autolysosome formation [35,38]. Therefore, p3a does not interfere with autophagosome formation or autophagosome–endosome fusion. Instead, it causes a specific defect of autophagosome/amphisome–lysosome fusion and blocks autolysosome formation (Figure 3). Notably, HeLa cells overexpressing ACE2 and infected with SARS-CoV-2 have protein dynamics similar to that in the cells expressing p3a (see above). In addition, the same is true for ultrastructural analysis, which showed the accumulation of autophagosomes and amphisomes in both p3a-expressing and SARS-CoV-2-infected cells [38,39]. SARS-CoV-2 infection also affected the size of autophagosomes, which were smaller in the infected cells. In conclusion, the SARS-CoV-2 3a protein can block autophagosome/amphisome–lysosome fusion when expressed by itself and in the context of SARS-CoV-2 infection.

Mechanistically, SARS-CoV-2 p3a co-localizes with late endosomes and this co-localization depends on its internal TM domains [35,38,39]. Then, via its C-terminal domain, p3a binds the homotypic fusion and protein sorting (HOPS) complex-specific subunit, VPS39 [26,38,39]. This p3a–VPS39 interaction affects the formation of a functional HOPS complex because it increases the association of VPS39 with the proximal subunits, VPS11 and VPS18, and decreases the association of VPS39 with the distal subunits, VPS16 and VPS33A, which form the synaptosome-associated protein (SNAP) receptor (SNARE)-binding subcomplex of the HOPS complex [38]. The HOPS complex plays an important role in mediating the fusion of autophagosomes/amphisomes with lysosomes by helping the SNARE complex of the autophagosomal SNARE, syntaxin 17 (STX17), lysosomal SNARE, vesicle-associated membrane protein 8 (VAMP8), and their bridging SNAP (SNAP29) to fuse the two membranes. In addition to assembly, HOPS complex localization is also affected in p3a-expressing cells: VPS39 and other subunits of the complex are recruited to late endosomes, despite decreased interaction of VPS39 with RAB7A [38,39]. Importantly, VPS39 is also recruited to late endosomes in SARS-CoV-2-infected cells. Due to such p3a-mediated re-distribution of HOPS complexes, starvation-induced autophagosomes are largely devoid of them and STX17 binds less of both SNAP29 and VAMP8, whereas SNAP29 binds less of VAMP8, in p3a-expressing cells [38,39]. Therefore, by sequestering the HOPS complex at the surface of late endosomes, SARS-CoV-2 p3a affects the formation of the *trans*-SNARE complex between autophagosomes/amphisomes and lysosomes, abrogating their fusion (Figure 3).

Interestingly, the negative effect of p3a on autophagy is SARS-CoV-2 specific. Although 3a proteins are highly conserved between the SARS-CoV and SARS-CoV-2 coronaviruses, SARS-CoV p3a cannot generate any autophagic defects [38,39]. Moreover, it was even reported that the SARS-CoV p3a oligomers cause activation and nuclear translocation of transcription factor EB (TFEB) that upregulates the mRNA and protein levels of the autophagy- (SQSTM1) and lysosome (LAMP1)-related genes. Such upregulation is probably a response to the lysosomal localization of SARS-CoV p3a and the damage it causes there [40]. Another exciting thing is that a single gene KD can rescue the autophagy defects created by the SARS-CoV-2 3a protein. Since O-linked N-acetylglucosamine transferase (OGT) suppresses the involvement of SNAP29 in the *trans*-SNARE complexes, *OGT* KD restores binding of STX17 to SNAP29 and VAMP8 in SARS-CoV-2 p3a-expressing cells with all the positive outcomes for autophagic flux, such as reductions in LC3 puncta, LC3-II and p62 levels, and a larger number of autolysosomes under both normal and starvation conditions [38]. To summarize, the depletion of the HOPS complex at the surface of autophagosomes/amphisomes by SARS-CoV-2 p3a can be counteracted by the depletion of the OGT enzyme that inactivates SNAP29 (Figure 3).

#### 2.2.7. Protein 7a Affects Lysosome Acidification

Similar to SARS-CoV-2 infection and p3a expression, p7a expression also disturbs the proteome of late endosomes [35]. Like p3a, p7a co-localizes with RAB9A-positive late endosomes and, to a lesser extent, with the trans-golgi network protein 2 (TGOLN2; also known as TGN46)-positive *trans*-Golgi network, resulting in its substantial fragmentation. While both proteins also prevent autophagosome turnover (see above), this is where their similarities end. In contrast to p3a, p7a affects the acidification of lysosomes [35]. Therefore, p3a (blocks the fusion with lysosomes) and p7a (blocks acidification of lysosomes) might have a synergistic adverse effect on autophagosome turnover during SARS-CoV-2 infection in human cells (Figure 3), which deserves further investigation.

#### 2.2.8. Polymorphism in Viral and Autophagic Genes

A few studies on SARS-CoV-2 discussed the polymorphism in viral and autophagy genes. First, it was reported that NSP6, in the more recent isolates of SARS-CoV-2, has the L37F mutation [41]. Cassone and colleagues predicted that L37 is right after TM1 on the cytosolic side of the ER membrane in the SARS-CoV-2 NSP6 protein that has seven TM domains. While the change of L37 to F37 might decrease the stability of the protein, adding phenylalanine to the existing constellation of seven aromatic residues nearby might increase the association of NSP6 with the ER membrane and affect autophagosome formation [41]. It will be interesting to see the effect of this mutation on autophagosome size and number, and virus replication in human cells. On the other side, the polymorphism in p3a does not affect its interference with autophagy [38]. The expression of the most common p3a mutant variants, Q57H and G251V, still causes increased levels of LC3-II and SQSTM1. Another study examined polymorphism in the host *ATG16L1* gene [42]. Dent and colleagues demonstrated that human colon cancer (HCT116) cells expressing different variants of ATG16L1, autophagy-proficient T300 and autophagy-deficient A300, have different levels of ACE2 and member 5 of the heat shock protein family A (HSPA5; also known as GRP78) proteins, which are both relevant for SARS-CoV-2 infection. However, further studies are necessary to test if *ATG16L1* polymorphism plays any role in SARS-CoV-2 replication.

#### 2.2.9. Clinical Relevance of Autophagic Markers

Finally, in clinical studies of COVID-19 patients, serum levels of autophagic proteins, such as LC3, SQSTM1, and BECN1, predicted the severity of COVID-19 disease [43,44]. Xia and colleagues showed that the decrease in circulating levels of LC3 (in patients of any age) and SQSTM1 (in patients up to 50 years old) is associated with the development of moderate-to-severe COVID-19 [43]. Specifically, the decrease in LC3 concentration below 5.5 ng/mL has a significant and independent predictive potential. Clinicians could use this marker for early hospital admission of some COVID-19 patients. The findings of previous studies in tissue culture might be able to explain these results in COVID-19 patients. SARS-CoV-2 infection leads to the accumulation of LC3-II and SQSTM1 inside autophagosomes and amphisomes (see above), making these proteins unavailable for secretion into the serum of COVID-19 patients. The intracellular accumulation is probably the reason why COVID-19 patients with more severe disease have decreased levels of these proteins in the serum. In contrast, there is a significant increase in BECN1 levels in the serum of infected people [44]. Moreover, BECN1 levels positively correlate with the key biochemical parameters associated with SARS-CoV-2 infection and severity of COVID-19 disease. Therefore, BECN1 could be used as another COVID-19 marker to determine the severity of the disease.

In conclusion, this section demonstrates that SARS-CoV-2 has extensive interactions with autophagy in human cells that might start as early as viral attachment to the cell surface. While some viral proteins induce the selective autophagy of mitochondria (M protein) or MHC-I molecules (p8), others inhibit autophagosome formation (NSP15) or block autophagosome turnover (p3a, p7a, and E) by affecting either autophagosome/amphisome–lysosome fusion (p3a) or lysosome acidification (p7a). The outcome of the infection for autophagy might depend on cell type and various genetic (such as gene polymorphism) and environmental factors, highlighting the importance of in vivo studies in model organisms, as well as clinical studies, especially because autophagic proteins can be used as both diagnostic and prognostic markers in COVID-19 disease.

### 2.3. MERS-CoV

**Figure 4:**
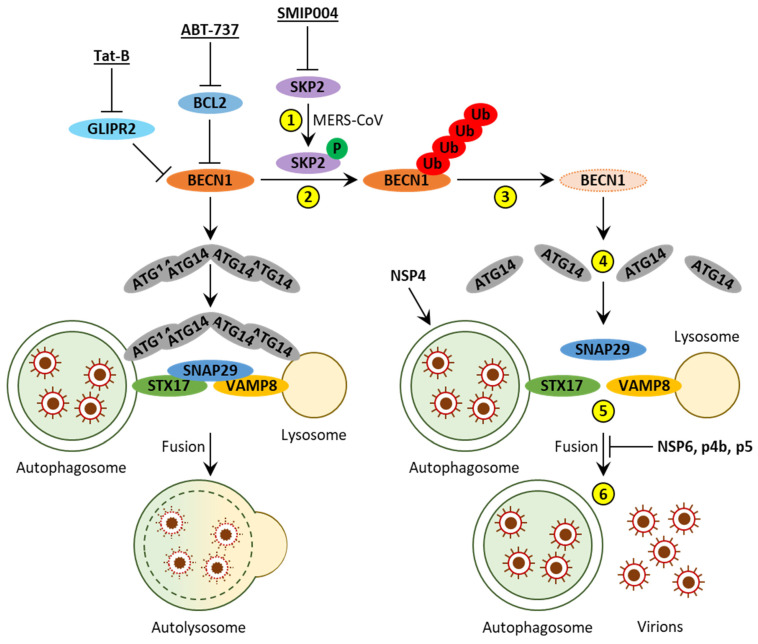
Interactions between MERS-CoV and autophagy. MERS-CoV induces autophagosome formation by NSP4 and blocks autophagosome–lysosome fusion by NSP6, p4b, and p5. The latter is accomplished via (yellow circles): (**1**) increased phosphorylation of SKP2, (**2**) enhanced polyubiquitination and (**3**) degradation of BECN1, (**4**) decreased ATG14 oligomerization, (**5**) reduced interaction of the autophagosomal SNARE, STX17, with the lysosomal SNARE, VAMP8, via the bridging protein SNAP29, leading to (**6**) reduced autophagosome–lysosome fusion and accumulation of autophagosomes and MERS-CoV virions. Inhibition of SKP2 with SMIP004 reverses these effects of MERS-CoV and decreases viral load. The same can be accomplished via other BECN1 preservation strategies, such as inhibition of the GLIPR2–BECN1 interaction with Tat-B or disruption of the BCL2-BECN1 binding with ABT-737. Protein and inhibitor symbols are in bold. Human proteins are in ovals. Inhibitor symbols are underlined. P, phosphate; Ub, ubiquitin.

The ORF1a of MERS-CoV also encodes a polyprotein that includes NSP3 with PLpro-TM domains. The transfection of this PLpro-TM coding sequence in HEK293T cells results in increased levels of LC3-II, similar to the transfection of SARS-CoV PLpro-TM (see above) [19]. Consistently, the infection of Vero B4 cells with MERS-CoV leads to increased levels of the autophagic receptor/substrate, SQSTM1, and a higher LC3-II/LC3-I ratio, indicating autophagosome accumulation. Treatment with the autophagosome–lysosome fusion inhibitor bafilomycin A1 does not further increase the LC3-II/LC3-I ratio, suggesting that this accumulation of autophagosomes is due to a fusion block. Indeed, the MERS-CoV infection of Vero B4 cells causes (Figure 4): (1) increased phosphorylation of S-phase kinase associated protein 2 (SKP2), (2) enhanced polyubiquitination and (3) degradation of BECN1 followed by (4) decreased ATG14 oligomerization, (5) reduced interaction of the autophagosomal STX17 with the lysosomal VAMP8 via SNAP29, leading to (6) a reduction in autolysosomes and accumulation of autophagosomes. While the KO of the *ATG5* gene causes a moderate increase in viral genomic RNA, it results in a strong increase in the number of viral particles [45]. Together, these results suggest that, similar to SARS-CoV/SARS-CoV-2, MERS-CoV can induce the accumulation of autophagosomes. This accumulation of autophagosomes is at least partially due to a block of the autophagosome–lysosome fusion, and this block of the fusion is beneficial for the virus, because it helps to preserve the assembled MERS-CoV virions (Figure 4).

The same study also showed that inhibition of SKP2 in MERS-CoV-infected Vero B4 cells results in (Figure 4): increased ATG14 oligomerization, enhanced interaction of STX17 with VAMP8 and SNAP29, leading to an increased number of autolysosomes, decreased number of autophagosomes, and a strong reduction in the levels of MERS-CoV genomic RNA [45]. Interestingly, inhibition of SKP2 in MERS-CoV-infected cells was not accompanied by a decrease in the ratio of LC3-II/LC3-I. However, treatment of these cells with both the SKP2 inhibitor SMIP004 and bafilomycin A1 did increase the LC3-II/LC3-I ratio further. These two results suggest a dual positive effect of SMIP004 on autophagosome formation and autophagosome–lysosome fusion. However, only the latter (increase in the number of autolysosomes) was confirmed by fluorescence microscopy, as mentioned above. In addition, this study revealed that any strategy that makes BECN1 more available to the autophagy pathway could potentially achieve similar antiviral results (Figure 4). Both (i) a BECN1-derived peptide, Tat-B, that binds GLI pathogenesis-related 2 (GLIPR2; also known as GAPR-1) and prevents sequestration of BECN1 by GLIPR2, and (ii) a BECN1 BH3 domain-like small molecule, ABT-737, that attaches to the apoptosis regulator, BCL2, and interferes with inactivation of BECN1 by BCL2, decrease the levels of SQSTM1 and increase the LC3-II/LC3-I ratio (which is consistent with autophagy induction) in Vero B4 cells, while decreasing the levels of viral RNA in MERS-CoV-infected Vero B4 cells [45].

Combined, the studies reviewed in this section suggest that MERS-CoV might be able to induce autophagosome formation (NSP4), while inhibiting autophagic flux by blocking the autophagosome–lysosome fusion (NSP6, p4b, and p5). Counteracting this fusion block with SKP2 inhibitors or BECN1 re-directing approaches might prove efficient in fighting MERS via enhanced clearance of MERS-CoV particles in human cells (Figure 4).

### 2.4. HCoV-NL63

**Figure 5:**
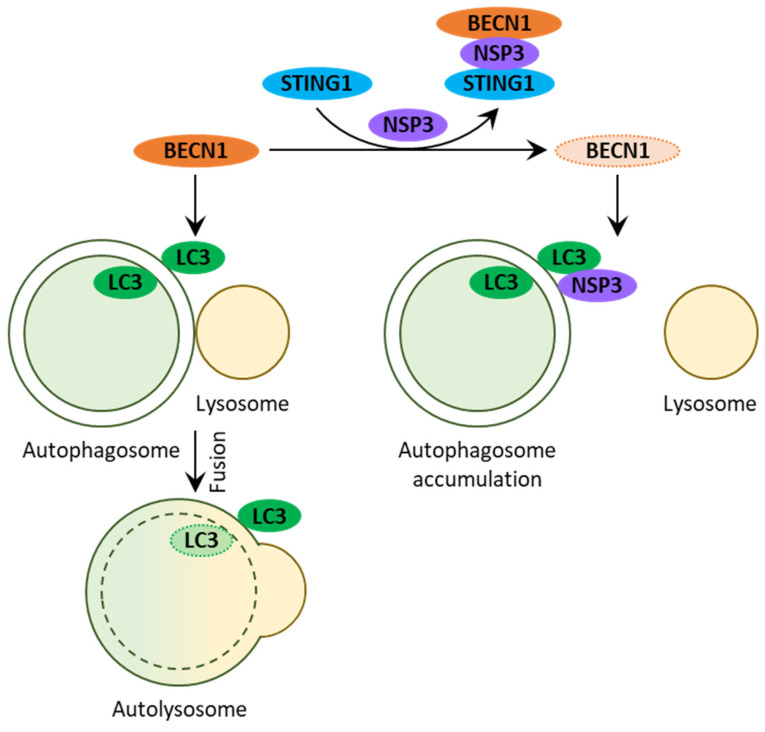
Interactions between HCoV-NL63 and autophagy. NSP3 of HCoV-NL63 blocks autophagosome–lysosome fusion. NSP3 co-localizes with autophagosomes and binds LC3 and BECN1 proteins. The latter interaction re-directs BECN1 to STING1 and makes it less accessible for autophagy. Protein symbols are in bold and in ovals.

Similar to previous coronaviruses, the ORF1a-encoded NSP3 of HCoV-NL63 contains papain-like protease 2 (PLP2) and TM domains. The ectopic expression of this PLP2-TM in HEK293T cells causes an increased: (1) level of LC3-II, (2) number of cells with LC3-positive puncta, and (3) number of double-membrane vesicles per cell, which is consistent with accumulation of autophagosomes [19]. Interestingly, a positive effect of PLP2-TM on LC3-II levels is independent of its catalytic activity. Moreover, it is not cell line specific either, because a similar increase in both the LC3-II levels and LC3 puncta was also observed in HeLa and breast cancer (MCF-7) cells transfected with PLP2-TM. The increased levels of SQSTM1 and accumulation of autophagosomes rather than autolysosomes suggest that PLP2-TM prevents autophagosome–lysosome fusion (Figure 5). Importantly, PLP2-TM of NSP3 might be doing this directly because it co-localizes with LC3-positive autophagosomes and physically interacts with LC3-II (more so than it does with LC3-I) and BECN1. The latter interaction is especially interesting, since the stimulator of interferon response cGAMP interactor 1 (STING1) can co-immunoprecipitate BECN1 only in the presence of PLP2-TM (which is also co-immunoprecipitated by STING1) [19]. This raises an interesting possibility that NSP3 of HCoV-NL63 might be blocking autophagosome–lysosome fusion by re-directing BECN1 to STING1 and making it less available for autophagy (Figure 5). Together, the studies on SARS-CoV-2, MERS-CoV, and HCoV-NL63 propose that the autophagosome–lysosome fusion block might be a common strategy of human coronaviruses to evade autophagy. However, they can achieve it by different means, such as sequestration of HOPS complexes (SARS-CoV-2) and degradation (MERS-CoV) or sequestration (HCoV-NL63) of BECN1 protein.

### 2.5. HCoV-OC43

Another mild coronavirus that is known to infect humans is HCoV-OC43. The infection of human lung fibroblast (MRC-5) cells with HCoV-OC43 initially results in higher levels of LC3-I expression that later translates into increased levels of LC3-II that correlate with an elevated number of SQSTM1 puncta [46]. However, the SQSTM1 protein does not accumulate in infected cells, suggesting that HCoV-OC43 causes increased autophagosome formation and not a block of the autophagosome–lysosome fusion. Interestingly, the inhibitor of HCoV-OC43 replication, kurarinone, increases the levels of LC3-II protein and SQSTM1 puncta even more, and also elevates the levels of SQSTM1 protein, indicating that this drug blocks autophagic flux after the autophagosome formation step. Therefore, while HCoV-OC43 induces autophagy, kurarinone inhibits both autophagy and HCoV-OC43 replication [46]. Without a doubt, the curious case of HCoV-OC43 deserves further studies to understand the physiological role of autophagy in HCoV-OC43 infection.

## 3. Conclusions

Seven human coronaviruses have been discovered to date. Some of them (HCoV-229E, HCoV-NL63, HCoV-OC43, and HCoV-HKU1) cause infection with mild symptoms, while others (SARS-CoV, SARS-CoV-2, and MERS-CoV) infect humans with more severe outcomes and can be life-threatening. Both mild and severe coronaviruses interact with autophagy, the conserved membrane trafficking pathway that operates in all eukaryotic cells and brings intracellular material from the cytoplasm to the lysosome for degradation and recycling. While formation of double-membrane vesicles, including autophagosomes, is beneficial for coronaviruses, the subsequent autophagosome–lysosome fusion is detrimental for them, as it results in the clearance of viral particles. Therefore, HCoV-NL63, SARS-CoV-2, and MERS-CoV evolved mechanisms that inhibit this fusion, leading to the accumulation of autophagosomes in infected cells. Interestingly, these mechanisms in HCoV-NL63 and MERS-CoV converge at BECN1, decreasing the availability of this protein for autophagy. The SARS-CoV-2 virus evolved a unique mechanism to block the autophagosome–lysosome fusion by sequestration of the HOPS complex. Since autophagosomes might serve as a suitable viral replication niche or the means to degrade detrimental host proteins and organelles, at least some coronaviruses (e.g., HCoV-OC43, SARS-CoV-2, and MERS-CoV) can induce their formation.

Given this crosstalk between human coronaviruses and autophagy, it is not surprising that many FDA-approved drugs, which are able to modulate autophagy, possess antiviral activity against human coronaviruses. Recently, several excellent reviews have been published on this subject [47,48,49,50,51]. As mentioned above, in some cases, the antiviral effects of the drugs correlate with their ability to inhibit autophagy [30,31,46]. However, in other cases, the antiviral activities of the drugs are linked to the upregulation of autophagy (e.g., niclosamide induces autophagosome formation, similar to MERS-CoV itself, but it also overcomes a block of the autophagosome–lysosome fusion caused by MERS-CoV, resulting in a spectacular clearance of the virus by autophagy [45]). Overall, the main take-home lesson that can be learned from the literature on human coronaviruses and autophagy is that human coronaviruses target autophagy for different purposes and autophagy plays a role in controlling their infection. Due to its antiviral capacity, autophagy is often blocked by human coronaviruses. To harness the full power of autophagy against coronaviruses, especially SARS-CoV-2, we need to fully understand the coronavirus–autophagy interplay. This will aid us in finding efficient ways to either remove (e.g., by targeting the SARS-CoV-2 p3a) or bypass (e.g., by targeting the host OGT) the virus-imposed autophagy blocks during coronaviral infections.

## Data Availability

Not applicable.
